# Deep Learning and Domain-Specific Knowledge to Segment the Liver from Synthetic Dual Energy CT Iodine Scans

**DOI:** 10.3390/diagnostics12030672

**Published:** 2022-03-10

**Authors:** Usman Mahmood, David D. B. Bates, Yusuf E. Erdi, Lorenzo Mannelli, Giuseppe Corrias, Christopher Kanan

**Affiliations:** 1 Department of Medical Physics, Memorial Sloan Kettering Cancer Center, New York, NY 10065, USA; erdiy@mskcc.org; 2 Department of Radiology, Memorial Sloan Kettering Cancer Center, New York, NY 10065, USA; batesd@mskcc.org; 3 IRCCS SYNLAB SDN S.p.A., 80143 Naples, Italy; mannellilorenzo@yahoo.it; 4 Department of Radiology, University of Cagliari, 09124 Cagliari, Italy; corriasmd@gmail.com; 5 Chester F. Carlson Center for Imaging Science, Rochester Institute of Technology, Rochester, NY 14623, USA; kanan@rit.edu

**Keywords:** deep learning, computed tomography, liver segmentation, artificial intelligence, image-to-image translation, dual energy computed tomography

## Abstract

We map single energy CT (SECT) scans to synthetic dual-energy CT (synth-DECT) material density iodine (MDI) scans using deep learning (DL) and demonstrate their value for liver segmentation. A 2D pix2pix (P2P) network was trained on 100 abdominal DECT scans to infer synth-DECT MDI scans from SECT scans. The source and target domain were paired with DECT monochromatic 70 keV and MDI scans. The trained P2P algorithm then transformed 140 public SECT scans to synth-DECT scans. We split 131 scans into 60% train, 20% tune, and 20% held-out test to train four existing liver segmentation frameworks. The remaining nine low-dose SECT scans tested system generalization. Segmentation accuracy was measured with the dice coefficient (DSC). The DSC per slice was computed to identify sources of error. With synth-DECT (and SECT) scans, an average DSC score of 0.93±0.06 (0.89±0.01) and 0.89±0.01 (0.81±0.02) was achieved on the held-out and generalization test sets. Synth-DECT-trained systems required less data to perform as well as SECT-trained systems. Low DSC scores were primarily observed around the scan margin or due to non-liver tissue or distortions within ground-truth annotations. In general, training with synth-DECT scans resulted in improved segmentation performance with less data.

## 1. Introduction

The automatic segmentation of the liver and associated tumors from single energy computed tomography (SECT) exams remains a challenge because of limited training data and overlapping intensity values of tissues or materials with different elemental compositions [1,2]. Most deep learning(DL)-based segmentation systems use object-level models that disregard the influence of tissues with different compositions (i.e., iodine-rich blood vessels or organs) [2,3]. Moreover, with SECT scans, it is technically challenging to identify or classify tissue composition strictly based on the intensity measurement or CT Hounsfield unit (HU) [1,3]. However, with dual-energy CT (DECT), the differential attenuation properties of tissues at low and high X-ray energies are exploited to differentiate and quantify material composition [1,3] and generate multiple image types. For example, DECT material density (MD) images display the concentration of specific elements such as iodine (MDI) throughout the scanned volume while suppressing any pixels with attenuation patterns, unlike iodine. DECT-based virtual monochromatic images (DECT-VMI) display anatomy from the viewpoint of a monochromatic X-ray source. Each of the image types provides a richer representation of the scanned anatomy and is reported to aid radiologists for specific diagnostic tasks [4,5,6,7,8]. However, the expensive cost of DECT capable scanners has limited their availability to academic medical centers [9,10]. Recent research efforts aim to broaden access to DECT technology by training artificially intelligent (AI) image-to-image translation systems to convert SECT scans into synthetic DECT (synth-DECT) image types that can then be used clinically by radiologists or medical centers that do not have dedicated DECT scanners [11,12,13,14,15,16,17,18,19,20]. The goals of the current image-to-image translation approaches are to infer DECT image types that radiologists can use for diagnosis. Instead, we hypothesize that AI systems trained on synth-DECT MDI MDI scans will enable generalization when working with limited data.

We test the hypothesis with a comparison study between AI systems trained with SECT and then again with the synth-DECT MDI scans to segment the liver from each respective patient CT scan. Similar to previous works [18,20], we train a 2D Pix2Pix conditional adversarial generator [21] to map SECT scans to synth-DECT MDI scans. The synthetic scans are then used to train four existing AI-based segmentation frameworks and their performance is compared with the same systems trained using the SECT scans. We find that AI systems trained on the synth-DECT MDI scans generalize better and with less data. We attribute the finding to the reduced overlap in image intensity values between different tissues and materials and the improved contrast between the target organ (i.e., liver) and the surrounding tissue in the synthetic images. In essence, DECT MDI image types provide clues about the diagnostic task because contrast-enhanced CT scans are designed to start precisely when the injected iodinated contrast is maximally concentrated in the target organ. Hence, the intensity of the target organ under investigation will be greater than the surrounding tissues with less iodinated contrast.

Our primary contributions are summarized as follows:

1. We define an image translation paradigm for creating synth-DECT MDI scans from SECT scans. This is performed by using co-registered DECT scan pairs to train a system that maps SECT scans to the synth-DECT MDI scans.
2. We study the benefits of using the synth-DECT MDI scans for liver segmentation in CT scans. We analyze their utility with four existing semantic segmentation algorithms. We found that the synthetic scans yielded superior performance over the original SECT scans when used as input.
3. We hypothesized that synth-DECT MDI scans would provide greater benefit when less training data were available compared to SECT scans, and we confirm that this hypothesis is generally supported in a study.
4. We additionally observed that the public dataset we used had distortions throughout the ground truth annotations of several scans, but the systems trained with the synth-DECT MDI scans correctly outlined the true extent of the liver for most scans, despite errors in the ground truth used for training.

## 2. Related Works

DL-based image to image translation to infer DECT image types: The feasibility of generating synth-DECT image types from SECT scan data using DL-based methods is reported throughout the literature [12,13,14,15,16,18,19,20,22,23,24,25,26,27]. These studies demonstrate how DL-based image translation methods can create synth-DECT scans for clinical interpretation. Recently, Seibold, C. et al. [28] trained existing image translation networks, such as Pix2Pix [21], to infer 40 keV DECT VMI images from SECT scan data acquired on a detector-based DECT scanner. The DL-based image translation frameworks were trained using paired source SECT scans and target domain DECT VMI images reconstructed at 40 keV. The resulting synt-DECT 40 keV VMI scans were then used to train a DL-based system to classify pulmonary emboli. However, the approach is enabled by the availability of paired 120 kVp SECT and spectral scan data from the detector-based DECT solution [28], which is unavailable for source-based DECT systems where the tube potential rapidly alternates between a low and high energy X-ray spectrum [29]. Our study consists of two parts where we first use co-registered or paired DECT VMI 70 keV and MDI scans to train a DL-based image-translation system to convert SECT scan data to synth-DECT MDI scans. Then, we demonstrate the improved performance of four existing DL-based liver segmentation systems when trained with the synth-DECT MDI scans relative to systems trained with SECT scan data.

## 3. Materials and Methods

**Figure 1:**
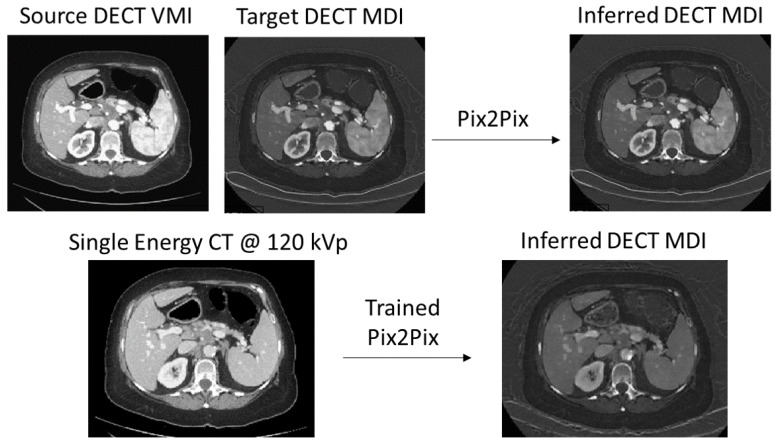
The Pix2Pix system was trained to map dual-energy CT virtual monochromatic images (DECT VMI) reconstructed at 70 keV to DECT material density iodine (MDI) images. Then, the trained system is used to convert single energy CT (SECT) scans acquired at 120 kVp to the synth-DECT MDI image types. Four liver segmentation frameworks were trained and tested with synth-DECT MDI and SECT scans.

An overview of our approach is shown in Figure 1. Section 3.1 describes how we trained and evaluated the Pix2Pix system to generate synth-DECT MDI scans. Section 3.2 describes the methods used to evaluate the usefulness of the synth-DECT MDI scans for training four different DL-based liver segmentation frameworks. For each section, we used two different datasets that are described below and summarized in Table 1. We use the first internal dataset to train the Pix2Pix network because it consists of paired image representations. However, it did not have pixel level annotations that outlined the liver. As a result, for the second part of this study where we train DL-based frameworks to segment the liver, we used the publicly available CT-ORG: CT volumes with multiple organ segmentation dataset [30,31] for which pixel level annotations were available.

Institutional review board approval was obtained for this Health Insurance Portability and Accountability Act-compliant retrospective study. The requirement for informed consent was waived. All data were collected retrospectively.

### 3.1. Generating Synth-DECT MDI Scans

In this subsection, we describe how we generated the synth-DECT MDI scans using a 2D Pix2Pix system. Pix2Pix is a conditional generative adversarial network (cGAN) that requires co-registered images with pixel-wise correspondence for training. With rapid switching DECT, paired SECT and DECT MDI image types are not available. However, the attenuation pattern observed on the DECT VMI 70 keV image is similar to SECT scans acquired with an X-ray energy of 120 kVp [9,32,33]. Due to the similarity, we used DECT VMI 70 keV scans as surrogates for the 120 kVp SECT scans. We only consider the cross-sectional axial views because the original coronal and sagittal reformats were not available.

To train Pix2Pix, we used 100 unique DECT patient scans for which paired reconstructions were available. The dataset was divided into a training, tuning, and test set, each of which had 80, 10, and 10 paired DECT scans, respectively. Each patient received a routine DECT scan between June 2015 to December 2017 to evaluate the liver. The scans were acquired on a 64 slice CT scanner (Discovery CT750 HD, GE Healthcare, Milwaukee, WI, USA) with rapid switching DECT following the intravenous administration of 150 mL of iodinated contrast (Iohexol 300 mgI/mL, Omnipaque 300, GE Healthcare, Cork, Ireland) at 4.0 mL/s. The scan parameters and patient characteristics are displayed in Table 1. The paired images used to train the Pix2Pix network were generated using the GSI MD Analysis software available on Advantage Workstation Volume Share 7 (GE Healthcare). For this study, no exclusion criteria were applied. All patients were included in the training stage.

To generate synth-DECT MDI scan types, we trained Pix2Pix to learn the transform between DECT VMI 70 keV and DECT MDI scans. We considered the slices of each DECT VMI 70 keV scan as the input domain, x∈X, that would be mapped to the DECT MDI image types in the output domain, y∈Y. For the generator, a 2D u-net was trained to learn a mapping from G:x→y by minimizing the difference between the paired DECT VMI and MDI slices. The objective of the input domain *x* and output domain *y* is expressed as follows:

(1) $$L_{cGAN}\left(G,D\right)\;=\;E_{x,y}[logD\left(x,y\right)+E_{x}[log\left(1-D\left(x,\;G\left(x\right)\right)\right],$$

where *G* is the generator loss that minimizes the objective against the discriminator *D*, which contrarily tries to maximize loss [21]. $E_{x,y}$ is the expectation with respect to the input and output, and $E_{y}$ is the expectation with respect to the output. As in the original Pix2Pix application, we use the L1 distance to mitigate blurring:

(2) $$L_{L1}=E_{x,y}[||y-{G\left(x\right)||}_{1}],$$

where $E_{x,y}[||y-{G\left(x\right)||}_{1}]$ is the average or expected value of the difference between the predicted output, y, and the generated image G(x). The final objective is as follows:

(3) $$G^{*}=arg\min_{G}\max_{D}L_{cGAN}\left(G,D\right)\;+\;\lambda L_{L1}\left(G\right)$$

where $G^{*}$ is the minimum with respect to G, the generator, of the maximum with respect to D, the discriminator, and λ is the learning rate. The architectures of the generator and discriminator include concatenated skip connections that learn low-level descriptors between the input and output. In addition, the discriminator uses PatchGAN, which penalizes structures at the scale of patch size.

#### 3.1.1. Implementation Details

Pix2Pix was trained for 100 epochs using an Adam optimizer with a learning rate of 0.0002, $\beta_{1}$ of 0.5, $\beta_{2}$ of 0.99, and weight decay of 0.000001. Since the framework expects a 3-channel image, each slice of a patient’s CT scan was copied into the red, green, and blue (RGB) channels to generate a faux RGB image. Because the input layer of the generator u-net was designed to accept 256×256 images, we resized each 512×512 CT scan to a dimension of 256×256 using bilinear interpolation. The generator part of the u-net is comprised of kernels with a size of 4×4 and a stride of 2 to downsample the input source up to the bottleneck layer. The decoder used transpose convolutions to upsample the original input image size. Skip connections were added between layers *i* and n−i, where n is the total number of layers. Each skip connection concatenates the channels at layer *i* with those in layer n−i to connect layers in the encoder to the corresponding layers in the decoder with the same sized feature maps. During training and inference, dropout is applied at a probability of 0.5, and batch normalization is used according to the respective train dataset statistics instead of the aggregate statistics of the training batch. A 3-layer PatchGAN with a patch size of 70×70 was used for the discriminator, along with a stride of 2 and kernel size of 4×4. Model weights were initialized using a random Gaussian with a mean of zero and a standard deviation of 0.02. These parameters are the defaults used to train the original Pix2Pix model. The remaining details are as specified in the original Pix2Pix paper [21].

#### 3.1.2. Image Preprocessing

The image preprocessing steps were similar to past studies in which similar datasets were used [34,35]. Since the voxel size varied from patient to patient, the DECT VMI and MDI scans were first resampled to an isotropic resolution of 1.0 × 1.0 × 1.0 mm using SINC interpolation. Then, each slice was resized to a height and width of 256 × 256 pixels using bilinear interpolation, which is the input size expected by Pix2Pix. The voxel HU value of the DECT VMI scans were clipped to be between ±300 HU and then normalized to have zero mean and unit variance (i.e., [0,1]). The threshold of ±300 HU was chosen because HU values outside of the range were not relevant for the liver or surrounding tissues. We did not clip the intensity values of the original DECT MDI image types, but each MDI image was normalized to have zero mean and unit variance. The image normalization process was performed separately for DECT VMI and MDI scans because the pixel value of the MDI scan reports the concentration of iodine in units of milligram per volume (mg/cc). The datasets were normalized by subtracting the mean and dividing by the standard deviation computed from the respective training dataset. The scans were then oriented into the left, anterior, and superior (LAS) orientation and were converted into a portable graphic network (png) 8-bit image from their 12-bit input formats. We did not apply any additional denoising because, as indicated in Table 1, the original scans were reconstructed with adaptive statistical iterative reconstruction, which is a denoising algorithm. The dimensions of the final synth-DECT MDI scans were $256\times 256\times n_{slices}$ with pixel intensity values that ranged from 0 to 255.

### 3.2. Semantic Segmentation Algorithms

Our goal is to evaluate the value of the synth-DECT MDI scans with four existing DL-based semantic segmentation systems. The four networks were chosen due to their success in organ segmentation:

1. Three-dimensional u-net with two residual connections [36,37]. This is the enhanced version of the u-net that includes parametric rectified linear units and residual units, which are known to improve training speed, mitigate the degradation issue of deep networks [38,39], and produce a network robust against variations in datasets [36].
2. SegResNet [40] without the variational autoencoder. This network uses ResNet [41] for the encoder section but includes group normalization, which divides channels into groups and normalizes within each group [42]. The grouping alleviates the limitations of batch normalization for small batch sizes [42].
3. Dynamic u-net (DynUNET) [43] is based on the full resolution architecture of nnUNet [44,45]. It was chosen because it achieved state-of-the-art performance on the LITS and MSD liver datasets [44].
4. V-Net [43,46] includes an encoder and decoder stage that learns residual functions at each stage. It produces outputs that are converted to probabilistic segmentations of the foreground and background by applying a soft-max function voxel-wise [46].

We implement each network as described in the associated references or using the default parameters defined by the Medical Open Network for AI (MONAI) [43]. Additional details about the architectures may be found in the associated references.

All models were trained from scratch. The loss for each model was the sum of the Sorensen DICE coefficient (DSC) score and cross-entropy loss.

(4) $$L_{total}=L_{dice}+L_{CE}.$$

We compute the dice loss for each sample in a single batch and then average over the batch.

(5) $$L_{total}=1\;-\;\frac{2}{J}\sum_{j=1}^{J}\frac{\sum_{i=1}^{I}G_{i,j}Y_{i,j}}{\sum_{i=1}^{I}G_{i,j}^{2}\;+\;\sum_{i=1}^{I}Y_{i,j}^{2}}-\frac{1}{I}\sum_{i=1}^{I}\sum_{j=1}^{J}G_{i,j}logY_{i,j}.$$

Training was completed using 3D patches of the input. The size of the patch was set to 32×32×32 for each network. Similarly to previous liver segmentation works [47,48], each system was trained for 1000 epochs using the Adam optimizer, with a learning rate of 0.0001, batch size of 2, β1 = 0.9, β2 = 0.99, and a weight decay factor of 0.000001. We implemented a sliding window approach for model inference where non-overlapping patches of size 64×64×64 iteratively moved over each slice of the input volume. The optimal window patch size was determined empirically [49].

#### Image Preprocessing

The intensity values of the synth-DECT MDI scans were clipped to be between 50 and 180 and then normalized to zero mean and unit variance. The SECT scans were processed similarly, but the intensity was clipped to be between 50 and 255. These values were determined empirically. No additional data augmentations were performed during training or testing of the liver segmentation networks.

### 3.3. Dataset Splits and Statistical Analysis

**Figure 2:**
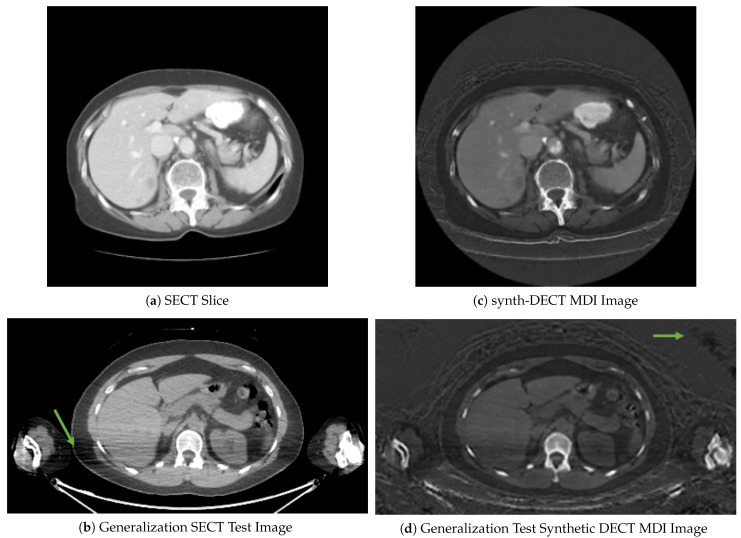
Cross sectional axial slices comparing the image-to-image translation for scans in CT-ORG. (**a**) A single axial slice from a patient single energy CT (SECT) scan. (**b**) Representative slice from one of the nine PET/CT scans used as the generalization test set: The streaks pointed to by the arrow are photon starvation artifacts that result from excess attenuation caused by the arms being at the side during the scan. (**c**) The synthetic dual energy CT material density iodine (synth-DECT MDI) image for the slice shown in (**a**). (**d**) The synth-DECT MDI image of the slice is shown in (**b**). The arrow in the synthetic slice shown in (**d**) points to a region in the air surrounding the patient that was distorted.

We divided the publicly available CT-ORG: CT volumes with multiple organ segmentation dataset [30,31] into a training and generalization test set. CT-ORG comprises of 140 SECT scans with detailed pixel-level annotations of the liver, lungs, bones, kidneys, and bladder. The first 131 scans and accompanying liver annotations are copied from two prior segmentation grand challenges, the Liver and Tumor Segmentation challenge (LITS) [45] and the medical Image Segmentation decathlon (MSD) [50]. These 131 SECT scans were used to train, tune, and test the four semantic segmentation frameworks. We only considered the liver annotations because the diagnostic task and delivery of iodinated contrast for the 131 SECT scans was optimized to visualize the liver and associated pathology. The remaining nine scans served as the test set for generalization assessment. They were suitable for evaluating system generalizability since they were low-dose, nondiagnostic attenuation correction CT scans. Apart from the fact that the nine scans were nondiagnostic, five of the nine patients had their arms placed at the side of the abdomen during the PET/CT. This contrasts with typical dedicated diagnostic CT scanning where patients raise their arms over their heads during the scan. As illustrated in Figure 2b,c, when the arms are positioned at the patient’s side during a low dose CT scan, the radiation dose is severely attenuated, resulting in multiple streak artifacts or dark and light bands that obscure the adjacent abdominal tissue.

Table 1 shows the scan parameters and patient characteristics that were made available with the dataset. Additional details about the CT-ORG dataset can be found in Rister et al.’s published report [30,45].

#### Statistical Analysis

The 131 scans were divided into five non-overlapping folds that consisted of 60% for training, 20% tuning, and 20% for the held-out test. Then, we performed stratified 5-fold cross-validation with the same division of scans across the four segmentation systems. The tuning dataset was processed every two epochs. We did not apply any additional data augmentation during training or testing.

We compare the performance of systems trained to segment the liver from SECT and then the synth-DECT MDI scans. The global DSC score was computed across each scan volume in the held-out and generalization test sets. The per-slice DSC score was also computed to identify the location of the errors in the scanned volume (i.e., presence of over or under-segmentation). The reported DSC scores reflect the average and standard deviation across the 5-fold cross-validation. We used the Mann–Whitney U test, with α=0.05, to calculate the significance of any observed difference between systems trained with the SECT and synth-DECT MDI scan types.

## 4. Results

### 4.1. Image Translation

We evaluate the quality of the mapping from DECT 70 keV VMI to the synth-DECT MDI scans using the held-out test set. To perform this, we compute the structural similarity index (SSIM) [51] between synthetic and original DECT MDI image types. SSIM is a metric that combines luminance, contrast, and structures into one index to assess the similarity between two images. We computed SSIM over the entire volume using MATLAB 2019b (version 9.7.0, Natick, MA, USA). We report the average and standard deviation of the SSIM across the held-out test cases used to assess the translation system.

**Figure 3:**
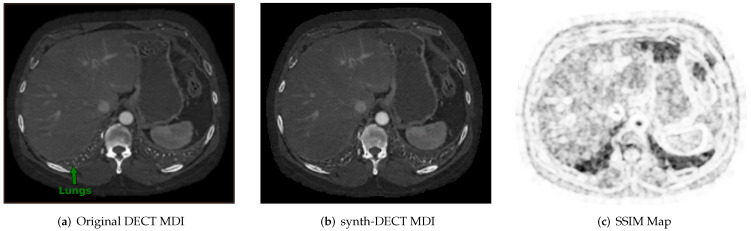
Example cross sectional axial slices from the test dataset used for Pix2Pix. (**a**) The original dual energy CT material density iodine (DECT MDI). (**b**) The synth-DECT MDI for the slice shown in (**a**). The global structural similarity index (SSIM) for the scan from which the slices were taken was computed to be 0.92. (**c**) This figure displays the local SSIM scores for each pixel of the slices in (**a**,**b**) as an image: The dark areas depict small values of the SSIM, which indicates a large difference between the original and synthetic image. The bright regions show large values of the SSIM or areas that were the most similar between the original and synthetic.

Across the nine test set scans, the average SSIM was computed as 0.94±0.014. Figure 3a,b shows an example cross-sectional axial slice from a single patient CT scan in the Pix2Pix test set. Subjectively, the original and synthetic slices in Figure 3a,b appear similar, but upon closer inspection, the base of the lung field pointed at in Figure 3a was blurred in the synthetic slice. Similar blurring in the lung field was observed across all test set scans. Figure 3c displays the local pixel level SSIM values computed between the slices shown in Figure 3a,b. The darker portions in Figure 3c point to air-filled cavities where the computed SSIM decreased. One reason for the low local SSIM within the air-filled cavities is that the effective attenuation of air within the lungs is neither similar to the two basis pairs, water or iodine, which were used to reconstruct the DECT image types. When the effective attenuation is unlike the two basis materials, a negative pixel value is assigned in the original DECT MDI scan.

The translation outcomes for two sample scans from the training and generalization test sets are shown in Figure 2. Subjectively, the anatomical structures are translated correctly. However, in the original SECT slices shown in Figure 2a,b, the bedding surrounding the patient seen in Figure 2c,d was not present. Because our objective was liver segmentation, the hallucinated bedding was excluded from subsequent tasks by first creating a binary mask of the body and then extracting only the pixels containing body information using the mask. The slices in Figure 2b,d are from a patient’s PET/CT scan in the generalization test set. The streaks indicated by the arrow in Figure 2b are due to the arms being down at the patients side and the use of a low dose CT scan. The synthetic counterpart shown in Figure 2d appears similar except for the distortions in the air surrounding the patient. Although distortions were evident in the synthetic slices, they reside outside of the body habitus; thus, they were not found to interfere with downstream tasks. With acceptable translation accuracy, we now evaluate our hypothesis that systems trained using the synth-DECT MDI scan types enable generalization with limited data.

### 4.2. Comparing SECT vs. Synth-DECT MDI Scans for Semantic Segmentation

#### 4.2.1. Main Results

The DSC score achieved by each system is shown in Table 2. On the CT-ORG held-out test set, the models trained with the synth-DECT MDI scans achieved a significantly higher average DSC of 0.93±0.06, whereas the models trained with SECT scans achieved an average DSC of 0.89±0.03, (p>0.001). As previously stated, the liver is expected to have the highest concentration and intensity of iodine. Thus, the improved performance of each system trained with synthetic scans could result from the improved contrast between the liver and background tissues. The performance of each model decreased on the generalization test set, but the systems trained with synth-DECT MDI scans outperformed those trained with SECT scans, as shown in Table 2. The gap in performance between the held-out and generalization tests could be due to the differences between the datasets. As discussed in Section 3.3, the CT portion of the PET/CT scan was not intended to be used by radiologists to make a primary diagnosis. Instead, the low-dose CT scan serves as an attenuation correction scan or is used to deliver enough radiation to outline the boundaries of the anatomy. Since the PET/CT scan time could be on the order of 20 min or greater, the arms are often placed at the patient’s side. Consequently, as shown in Figure 2b,d, the additional attenuation of the arms causes streak artifacts that obscure parts of the liver and adjacent abdominal organs.

#### 4.2.2. Performance with Increasing Training Set Size

**Figure 4:**
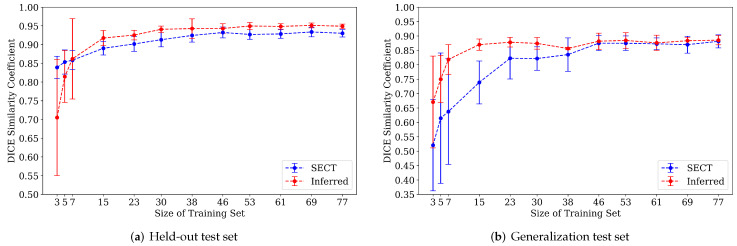
Comparison of segmentation accuracy (DICE) of liver vs. training set size. Average and standard deviation of the DICE score across 5-fold cross validation runs for the (**a**) held-out and (**b**) generalization test sets.

We hypothesized that the synth-DECT scans would provide greater benefit when the size of the training dataset was small. To test this hypothesis, we used the best performing system from our main results: the 3D u-net. The DSC score on the held-out and generalization test sets as a function of training set size for the 3D u-net is shown in Figure 4. The test set did not change as the training set size increased. As shown in Figure 4a, with 46 scans in the training set, the DSC score plateaued at 0.92±0.01 and 0.95±0.06 on the held-out test set for the systems trained with the SECT and synth-DECT MDI scans. On the generalization test set shown in Figure 4b, with 46 scans in the training set, the system trained with SECT scans achieved a DSC score of 0.83±0.01, and when trained with synthetic scans, the DSC score was 0.89±0.01.

#### 4.2.3. Failure Mode Analysis

**Figure 5:**
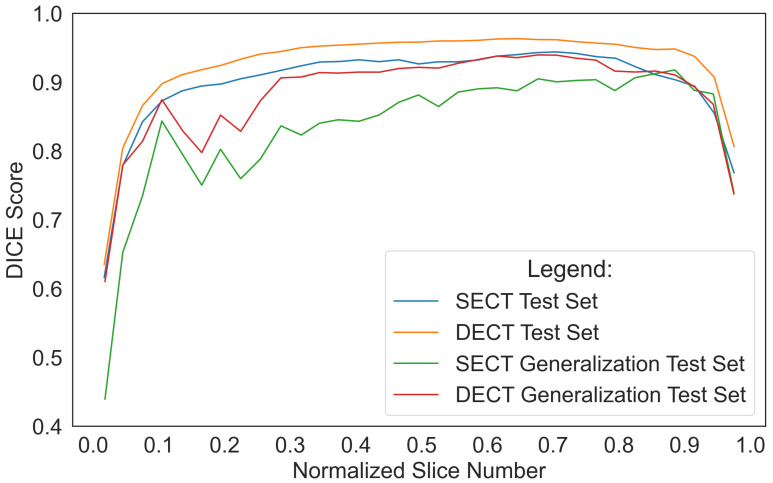
DICE Score per Slice. Line plot shows the normalized DICE score per slice for all scans in the single and synthetic dual energy CT (SECT; DECT) held-out and generalization test sets. The largest errors by the 3D u-net were at the beginning and end of each test scan.

To determine the source of the 3D u-net’s lowest DSC scores, we computed the DSC score per slice for each scan in the held-out and generalization test sets. Figure 5 shows the distribution of the DSC score per slice normalized by slice number for each scan in the SECT and synth-DECT MDI held-out and generalization test sets. For the SECT and synth-DECT MDI versions of the held-out test set, the DSC score fell below 0.90 along the first and last 10% of the slices in each scan. Similarly, on the generalization test set, the DSC score per slice decreased to less than 0.90 in the first 30% and last 10% of each scan, respectively.

**Figure 6:**
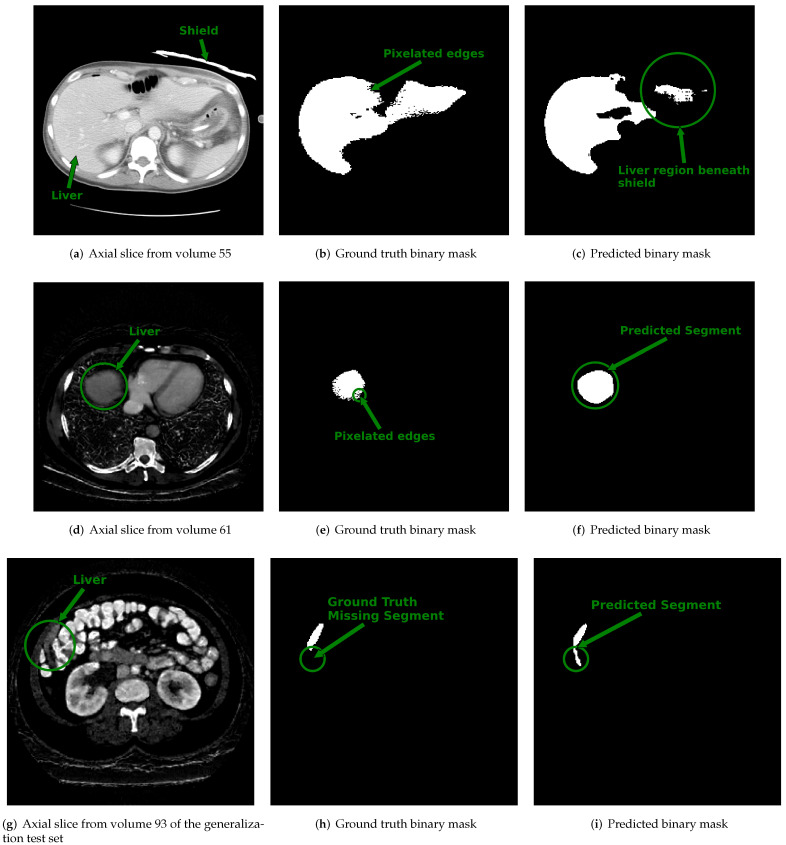
Displayed are example cross sectional axial slices with ground truth annotations and predicted contours from 3D u-net. **Top Row:** (**a**) Axial slice from single energy CT (SECT) scan of a patient within the CT-ORG training dataset shows an attenuating shield placed over segment 2 of the liver. (**b**) The ground truth binary image provided for the slice shown in (**a**) has pixelated edges pointed to by the arrow. (**c**) The output predicted by the 3D u-net for the slice in (**a**). The circled region pointed to by the arrow shows the area under the shield where the 3D u-net under-segmented the liver. **Middle Row:** (**d**) The synthetic dual-energy CT (synth-DECT) material density iodine (MDI) slice from a patient in the held-out test set. The liver is circled and pointed to by an arrow. (**e**) The ground truth binary image provided with the CT-ORG dataset for the slice shown in (**d**) also has pixelated edges that are circled and pointed to by the arrow. (**f**) The output predicted by the 3D u-net is circled and pointed to by the arrow. It incorporated the entire extent of the liver, without any pixelation. **Bottom Row:** (**g**) An axial slice from a patient scan in the generalization test set. The circled area and arrow point the portion of the liver at the margins of the liver. (**h**) The ground truth slice for the image shown in (**g**) does not contain a portion of the liver. The circle and arrow point to the segment of the liver missing from the ground truth annotation. (**i**) The predicted output by the 3D u-net. The circle and arrow point to the segment of the liver that was successfully identified by the 3D u-net, but was missing from the ground truth annotation shown in (**h**). The top row shows the impact of noise and beam hardening arising from the shield’s on the predictions of the 3D u-net. Several scans in the training dataset had ground truth contours with pixelated edges, missing segments of the liver, or inclusion of non-liver tissue, as shown in this figure.

Examples of slices from scans within the dataset with the lowest DSC values (i.e., DSC < 0.8) are displayed in Figure 6. Figure 6a shows the center slice of the liver, which is where the liver occupies around 50% or more of the abdominal space. In contrast, at the start and end slices, the liver tissue occupies a minor proportion of the abdominal area, as illustrated in Figure 6d,g. We suspect that the reduced DSC scores at the start and end slice locations are a byproduct of the small size of the liver tissue relative to the background and partial volume averaging artifacts that falsely reduce or increase the pixel intensity value of border pixels. Consequently, the class imbalance and artifacts at the margins of the scan may increase the likelihood of misclassifying pixels.

Moreover, each pixel intensity value in the synth-DECT MDI scans was transformed based on the amount of iodinated contrast it possessed. Iodine-rich pixels were brighter, whereas iodine-depleted pixels were less intense. As a result, the edges or boundaries of the liver tissue in the synth-DECT MDI scan types were improved. The improved boundary delineation explains why the performance of the 3D u-net trained with the synth-DECT MDI scan types outperformed that of the SECT scans in Figure 5.

Additional factors that contributed to the lower DSC score are also illustrated in Figure 6. In Figure 6a, we found a case in which a bismuth or lead shield was placed over the patient’s abdomen during the scan. The shield attenuates X-rays, causing beam hardening and streak artifacts, as well as increasing noise in the organs beneath it. In addition to the shield, the ground truth annotation provided by the dataset organizers shown in Figure 6b contained pixelated edges. As shown in Figure 6c, the combined effect caused the 3D u-net to undersegment the portion of the liver directly under the shield. Figure 6d–f show an example slice with its ground truth contour that contains pixelated edges and the predicted output of the 3D u-net. In this case, the reduced DSC score was not a result of over or under segmentation by the 3D u-net but was, instead, due to the differences arising from the pixelation in the ground truth and lack thereof in the predicted output. In another example shown in the final row of Figure 6g–i, the reduced DSC score for this case was because the ground truth annotation displayed in Figure 6h did not outline the entire segment of the liver. However, as illustrated in Figure 6i, the predicted output of the 3D u-net included the full extent of the liver. Several scans in the CT-ORG dataset had ground truth annotations that were rough outlines of the liver or consisted of pixelated edges [45]. Despite imprecise ground truth contours, the 3D u-net trained using synth-DECT MDI scans was still able to predict the complete extent of the liver tissue for many patient scans.

## 5. Discussion

This paper develops a method to generate synth-DECT MDI scans and demonstrates the benefits of using them to train neural networks for liver segmentation. Furthermore, we show that the 3D u-net trained with synth-DECT scans surpasses the performance of the same system trained with the SECT scans when less training data are available. We also found that the systems trained with synthetic scans were less susceptible to distorted annotations and their performance at the margins of the scan was better than the system trained with the SECT scans. The reduced performance at the margins of the scan may be due to a combination of factors, such as partial volume artifacts and class imbalance. The former could be addressed by scanning with smaller voxel dimensions [3] or by resampling scans into smaller voxel dimensions during the preprocessing steps. The latter could be addressed by implementing a class balancing scheme according to the pixel-wise frequency of each class in the dataset [52]. Since the goal of the current paper was to assess the value of synth-DECT scans, we did not implement class balancing schemes to mitigate the errors found at the margins of the scans.

The precise mapping of a SECT scan to an synth-DECT MDI scan type could also enable the possibility of realizing the benefits of DECT at institutions without DECT scanners. However, the influence of clinical variables such as the type of DECT scanner, patient size, position, iodine content, and scan parameters could dictate the quality and accuracy of the synthetically generated DECT scans [29,53]. For example, the internal data we used to train the Pix2Pix system were acquired with a rapid kVp switching DECT scanner. The tube potential rapidly alternates between the high and low-energy X-ray spectra with this DECT scanner. Due to the finite switching time and detector temporal response, some of the detected signals from the low and high energy spectra could overlap [29]. As a result, noise increases in the material decomposition images, and the quantitative accuracy reduces [29]. Since the tube current for the lower energy spectra of the rapid kVp switching DECT variant remains fixed, photon starvation artifacts and increased noise are commonly observed in patients who weigh more than 250 pounds or in scenarios where the arms cannot be raised above a patient’s head for body exams [29,54]. The impact of noise on the proposed method was observed in Figure 6d, where a shield placed over the abdomen attenuated X-rays, which then increased noise throughout the organs under the shield. Consequently, the proposed method undersegmented the portion of the liver that was under the shield. An additional factor that impacts the accuracy of material decomposition images is the iodine content within the target organs. As Corrias et al. [53] described, the iodine content may be influenced by patient characteristics or institutional scanning practices. For example, BMI strongly affects the timing of post-contrast enhancement of a target organ [53]. Hence, if the scan start time after contrast administration is not catered to the patient characteristics, the iodine concentration depicted on DECT MDI images may not be optimally distributed. As a result, the perceived difference between the target organ and the background tissue could be reduced. The reduced contrast may cause the proposed framework to undersegment or oversegment the liver. Since we used pre-existing datasets to train and test the proposed method, we could not control the variables described above. However, our study provides a proof of concept that demonstrates the improved performance of DL-based systems trained with synth-DECT MDI scans for liver segmentation.

Failure mode analysis showed how scanning practices and dataset quality issues could impact the proposed method. Training medical-grade AI systems with imprecise ground truth annotations could cause misdiagnosis. Including nonliver tissue increases the risk of learning to correlate features unrelated to the target task with the class labels. As a result, systems presumed to be working would fail to generalize when used clinically, or they would appear to be working, but for the wrong reasons [55]. In addition to stricter quality control standards and reporting criteria for training datasets, we identify the need for medical institutions’ to acceptance test or evaluate AI systems before they are used on patients. Acceptance testing would include evaluation with anthropomorphic phantom images or sample patient scans that are unique to the institution. The phantom images would provide an opportunity to understand the effect of the scanner settings. One must evaluate the AI systems’ generalization ability with institution-specific patient scans because local scanning practices and scanner technology may differ significantly from the training dataset. The goal would be to understand the limitations of the AI system and identify where or when it fails to perform the intended task. In addition, we encountered some limitations. The size and composition of our generalization test set were limited. More diverse test sets are needed to determine the full potential of our approach. Our investigation was also limited to liver segmentation. We did not investigate the ability of the system to separate tumors from the surrounding tissue, but we leave that investigation open for future work.

## 6. Conclusions

AI systems continue to grow in complexity and applications. Clinically reliable and trustworthy AI systems have yet to gain mainstream adaptation. Considering the imprecise ground truth annotations throughout the training dataset, we recommend more rigorous quality control standards that include a comprehensive verification of dataset annotations, including scan parameters within the meta-data, and identifying and reporting artifacts in scans. In conclusion, we exploited the diagnostic task, human physiology, and medical imaging physics to generate synth-DECT MDI scans that improved the performance of the tested liver segmentation systems with limited datasets.

## Figures and Tables

**Table 1:** Scan parameters and patient-specific characteristics for the datasets used to train the Pix2Pix system and then the semantic segmentation systems.

	Pix2Pix	Liver Segmentation
	Internal Data	Public Data
Pixel Annotations	No	Yes
CT Vendor	General Electric	**
CT Model	HD750	**
Total # Patients	100	140
# Used for Train	80	79
# Used for Val	10	26
# for test	10	26
Average age (min to max)	59 (18 to 88)	**
Scan start time after contrast administration	30 to 35s	**
Range of slices (min/max)	32 to 94	42 to 1026
Tube potential (kVp)	120	**
Slice thickness (mm)	2.5	0.45 to 6.0 mm
Pixel dimensions (mm)	0.606 to 0.977	0.56 to 1.0 mm
Tube current modulation index	NA	**
Tube current range	260 to 600 mA	**
Rotation time (s)	0.7	**
Pitch	0.984	**
Reconstruction algorithm	FBP *	**
Reconstruction kernel	Standard	**
Iterative reconstruction strength	20% ASiR ***	**
# of data channels	64	**
Size of a single data channel (mm)	0.625	**
Bowtie filter	Large Body	**

* Filtered Back Projection. ** Not available in accompanied report. *** Adaptive Statistical Iterative Reconstruction.

**Table 2:** Dice scores from the 5-fold cross validation and the nine test cases from the CT-ORG generalization dataset.

	Held Out Test Set		Generalization Test Set	
Model	Single Energy CT	Single Energy CT	SECT	Synthetic
3D u-net	0.92 ± 0.01	0.95 ± 0.06	0.83 ± 0.01	0.89 ± 0.01
SegResNet	0.89 ± 0.02	0.94 ± 0.01	0.88 ± 0.02	0.89 ± 0.01
DynUNET	0.89 ± 0.01	0.90 ± 0.01	0.82 ± 0.03	0.86 ± 0.01
VNET	0.89 ± 0.01	0.93 ± 0.01	0.85 ± 0.02	0.88 ± 0.01

## Data Availability

Restrictions apply to the availability of the DECT data used to train the image-to-image translation system. The DECT data are not available due to institutional policy. The CT-ORG data are publicly available: https://wiki.cancerimagingarchive.net/display/Public/CT-ORG%3A+CT+volumes+with+multiple+organ+segmentations, accessed on 16 July 2021.
